# Risk Assessment of *Spodoptera exempta* against Food Security: Estimating the Potential Global Overlapping Areas of Wheat, Maize, and Rice under Climate Change

**DOI:** 10.3390/insects15050348

**Published:** 2024-05-13

**Authors:** Ming Li, Zhenan Jin, Yuhan Qi, Haoxiang Zhao, Nianwan Yang, Jianyang Guo, Baoxiong Chen, Xiaoqing Xian, Wanxue Liu

**Affiliations:** 1 State Key Laboratory for Biology of Plant Diseases and Insect Pests, Institute of Plant Protection, Chinese Academy of Agricultural Sciences, Beijing 100193, China; 2 Western Agricultural Research Center, Chinese Academy of Agricultural Sciences, Changji 831100, China; 3 Rural Energy and Environment Agency, Ministry of Agriculture and Rural Affairs, Beijing 100125, China

**Keywords:** *Spodoptera exempta*, MaxEnt model, global warming, agricultural crops, risk assessment, potential overlapping areas

## Abstract

**Simple Summary:**

*Spodoptera exempta* is a global agricultural invasive pest which posing a serious threat to the planting area of major crops of maize, wheat and rice. Our study predicted the potential suitable areas for *S. exempta* globally based on 841 global occurrence records and eight important bioclimatic variables, and analyzed the overlap with maize, wheat and rice planting areas. The results of the study indicate that the high potential suitable areas of *S. exempta* are mainly distributed in developing countries (Latin America, central South America, Central Africa, and southern Asia), with the greatest impact on global maize planting areas, followed by rice and wheat. Global warming will have a smaller limitation on the distribution of *S. exempta*. In view of the global potential geographic distribution of *S. exempta* and its damage to three major crops, the above governments of various countries should pay active attention to the prevention and control of *S. exempta* to protect global food security.

**Abstract:**

*Spodoptera exempta*, known as the black armyworm, has been extensively documented as an invasive agricultural pest prevalent across various crop planting regions globally. However, the potential geographical distribution and the threat it poses to host crops of remains unknown at present. Therefore, we used an optimized MaxEnt model based on 841 occurrence records and 19 bioclimatic variables to predict the potential suitable areas of *S. exempta* under current and future climatic conditions, and the overlap with wheat, rice, and maize planting areas was assessed. The optimized model was highly reliable in predicting potential suitable areas for this pest. The results showed that high-risk distribution areas for *S. exempta* were mainly in developing countries, including Latin America, central South America, central Africa, and southern Asia. Moreover, for the three major global food crops, *S. exempta* posed the greatest risk to maize planting areas (510.78 × 10^4^ km^2^), followed by rice and wheat planting areas. Under future climate scenarios, global warming will limit the distribution of *S. exempta*. Overall, *S. exempta* had the strongest effect on global maize production areas and the least on global wheat planting areas. Our study offers a scientific basis for global prevention of *S. exempta* and protection of agricultural crops.

## 1. Introduction

*Spodoptera exempta* (Spodoptera; common name: black armyworm) is a highly aggressive agricultural invasive pest found across various global regions such as Sub-Saharan Africa (Kenya, Tanzania, South Africa, and the Arabian Peninsula), Oceania (Australia, Hawaii, and the USA), and South Asia (Indonesia, Kalimantan, Sulawesi, and the Philippines) [1,2,3]. The early records of *S. exempta* date back to the 16th century in Ethiopia, Hawaii in 1873, and South Africa in 1878, and occurrence records can be traced in Australia and most African countries until the 20th century; it was introduced to India after 1950 [2,4]. *Spodoptera exempta* primarily inflicts harm upon plants belonging to the families Poaceae and Cyperaceae, encompassing economically significant cultivars like barley, maize, oats, rice, sorghum, sugar cane, and wheat [5]. Heavy feeding on the leaves and young stems of host crops by *S. exempta* can lead to total defoliation of the crop and may damage the quality of the soil, thus reducing crop yields. For instance, during outbreaks in East Africa, *S. exempta* thrives at high larval densities, leading to reduced agricultural crop production and the degrading of rangelands [6]. In Zambia, invasion by *S. exempta* caused a decline of 11% maize production in 2013 and a more substantial reduction ranging from 30 to 40% in 2017 [7]. The adult stage of *S. exempta* has the ability to disperse naturally, primarily through migratory flight, while eggs and larvae can inadvertently spread via the unintentional dispersal associated with plants, wood products, or trade activities involving host crops [8]. With the rapid development of global economic integration and trade, *S. exempta* will be further spread [9]. However, studies on the potential geographic distribution of *S. exempta* around the world have not been carried out, which poses a significant challenge to the early warning, control and management of the species [7].

Ecological niche models (ENMs) are based on the theory of ecological niches and use mathematical models to simulate and generalize species habitat minimum thresholds and project them onto known environmental variables to make predictions about the potential geographic distribution of species [10]. Ecological niche models can be broadly categorized into two groups: correlative models and process-based models or mechanistic models [11]. The correlation model is represented by the MaxEnt model and the mechanistic model by the CLIMEX model (also an ecophysiological model) [12,13]. Because distribution data for species are easily available compared to biological parameters, correlation models are used in more species [14]. Among them, MaxEnt is one of the most commonly used correlation models [15], which can maintain a highly stable performance and demonstrates good prediction accuracy even with a small sample size [16,17]. It exhibits superior performance in analyzing the potential geographical distribution of agricultural invasive pests. For instance, previous studies have used MaxEnt to assess the global potential geographic distribution of four *Diabrotica* species and the risks for agricultural production [18]. However, the default-based model is prone to overfitting, leading to inaccurate prediction; therefore, we used the ENMeval package in R software (version 4.2.2) [19].

In order to clarify the potential geographical distribution of *S. exempta* in the world, and the harm it causes to the staple food areas, we used the MaxEnt model based on optimized parameters to predict the global potential suitable areas of *S. exempta* under current and future climate conditions and overlapped them with global planting areas of wheat, rice, and maize. Thus, (1) we established global potential suitable areas of *S. exempta* under current and future climate conditions, (2) we overlaid potential suitable areas of *S. exempta* with major planting areas of wheat, rice, and maize crops, and (3) we identified important bioclimatic variables that influence the global potential suitable areas of *S. exempta*. The resolution of these questions contributes to the understanding of the potential suitable areas of *S. exempta* and the damage to major crops, providing a further scientific basis for the global prevention of *S. exempta* and crop protection.

## 2. Materials and Methods

### 2.1. Occurrence Records of Spodoptera exempta

**Figure 1:**
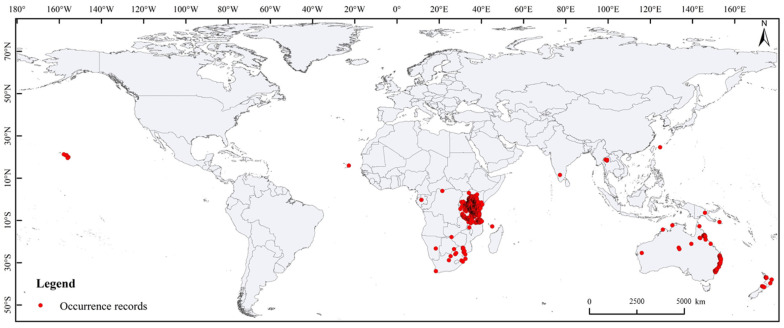
Global occurrence records of *Spodoptera exempta*.

The global occurrence records for *S. exempta* were sourced from the Global Biodiversity Information Facility (GBIF; https://www.gbif.org/, accessed on 18 November 2022), iNaturalist (https://www.inaturalist.org/, accessed on 18 November 2022), and the literature records. The occurrence record bias obviously affects the accuracy of model prediction results. First, the occurrence records without detailed information were removed; second, the remaining occurrence records were cleaned using the ENMtools software (version 1.1.2) to ensure that only one occurrence record was retained for each raster (5 arc minutes) [20]. After this process, 841 records were retained for MaxEnt model construction (Figure 1). This study used the MaxEnt model of version 3.4.1 (http://biodiversityinformatics.amnh.org/open_source/maxent/, accessed on 17 November 2022).

### 2.2. Bioclimatic Variables and Crop Data

In this study, 19 bioclimatic variables were selected (Table S1) (https://worldclim.org/data/bioclim.html). The current and future bioclimatic variables were sourced from the World Climate Database 2.0 (http://www.worldclim.org/) with a resolution of 5 arc minutes, and future bioclimatic variables were selected from the BCC-CSM2-MR climate system model developed by the National Climate Center, using two shared socioeconomic pathways (SSPs) (Table S2). Future climate data based on the BCC-CSM2-MR model performed well on both global and regional scales, For example, QBO, the MJO, the diurnal cycle of precipitation, interannual variations in SST in the equatorial Pacific, and so on [21]. The SSP1-2.6 scenario represents the combined effects of low vulnerability, low mitigating stress, and low radiative forcing; the SSP5-8.5 scenario represents the high forcing scenario [22]. Crop planting area data (wheat, rice, and maize) were sourced from the Spatial Production Allocation Model (https://www.mapspam.info/), with a resolution of 5 arc minutes.

Correlation analysis of 19 bioclimatic variables was performed using ArcGIS 10.7 software due to the presence of multicollinearity between the bioclimatic variables (Figure S1). If the value of the correlation coefficient of two bioclimatic variables was greater than 0.8 (|r| > 0.8), the one with the greater contribution of bioclimatic variables was retained. Finally, eight bioclimatic variables were retained for MaxEnt model construction: the mean diurnal air temperature range (Bio2), the temperature seasonality (Bio4), the max temperature of warmest month (Bio5), the precipitation of wettest month (Bio13), precipitation of driest month (Bio14), the precipitation seasonality (Bio15), the precipitation of warmest quarter (Bio18), and the precipitation of coldest quarter (Bio19).

### 2.3. Optimize the MaxEnt Model

Using the default parameters in constructing the MaxEnt model might lead to overfitting of the model prediction results [23]. Therefore, we used the ENMeval package in R software to adjust the two important parameters of the feature combination (FC) and regularization multiplier (RM) in MaxEnt [24]. The MaxEnt model provides five features: linear-L, quadratic-Q, hinge-H, product-P, and threshold-T. In this study, RM was set to 0.5–6, increasing by 0.5 each time, and six feature combinations were used [25]. AICc (Akaike Information Criterion Correction, AICc) is a mathematical method for evaluating the fit of a model to the data that produced it [26,27]. We used the ENMeval package in R software to optimize the model parameters, and we determined the model parameters according to the minimum AICc value, when model fitting was the lowest and prediction was the most accurate [28].

### 2.4. Model Construction and Suitable Area Classification

The global occurrence records of *S. exempta* and bioclimatic variables were imported into the MaxEnt model; 75% of the occurrence records were randomly selected as the training set, and the remaining 25% of the occurrence records were used as the testing set [29]. The parameters of the MaxEnt model were set according to the optimized feature combination, while the background points were set to 10,000, the maximum number of iterations was set to 500, the output format was Cloglog, and the runs were repeated 10 times for cross-validation. The receiver operating characteristic curve (ROC curve) was drawn with ‘1-specificity’ (false positive rate) as abscissa and sensitivity as ordinate. The area under ROC curve (AUC) was calculated as the test standard of model prediction accuracy. The value range of the AUC was 0–1, and a larger value indicates a higher model prediction accuracy, below 0.5 indicates model prediction failure, 0.5–0.8 indicates usable, and above 0.8 indicates excellent model prediction [18].

The results indicated the global probability of the presence of *S. exempta* with a value range of 0–1, with higher values indicating higher probability of species presence. We selected the maximum test sensitivity plus specificity Cloglog threshold in the MaxEnt model as the potential suitable area threshold for *S. exempta* worldwide. The model results were imported into ArcGIS software using the reclassify tool to classify the potential suitable areas for *S. exempta* into four kinds: 0–0.1838 was an unsuitable area, 0.1838–0.4 was a poorly suitable area, 0.4–0.6 was a moderately suitable area, and 0.6–0.1 was a highly suitable area. We used the raster calculator of ArcGIS software to overlap the potential suitable areas for *S. exempta* under current and future climatic conditions with the crop planting areas data to predict the area of damage to the host crop (wheat, maize, and rice) by the species.

## 3. Results

### 3.1. Evaluation of Model Performance

**Figure 2:**
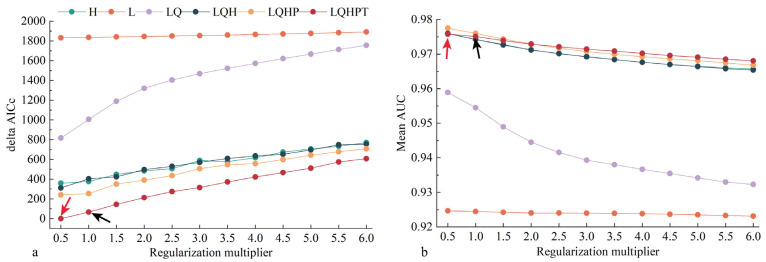
*Spodoptera exempta* modeling results as calculated using the ENMeval package, showing that the optimal parameter combination of FCs and RM in the MaxEnt models was LQHPT and 0.5. (**a**): Delta AICc value; the smaller the value the lower the model fit. (**b**): Mean AUC value; the optimized feature parameter has a higher value than the default feature parameter. The black arrows correspond to the default feature parameters, and the red arrows are the optimized feature parameters.

Overall, we obtained 72 different MaxEnt parameter combinations using 841 occurrence records and eight bioclimatic variables for *S. exempta*. The default settings for the MaxEnt model were FC = LQHPT, RM = 1 and delta AICc = 50.27. However, the optimized MaxEnt model revealed FC = LQHPT, RM = 0.5, and delta AICc = 0 (Figure 2). Following 10 repeated runs, the mean AUC value was 0.964 in the optimized MaxEnt model (Figure S2), indicating the optimized model has better fitting and mobility, and reduces the over-fitting of the model.

### 3.2. Important Bioclimate Variables and Response Curve

**Figure 3:**
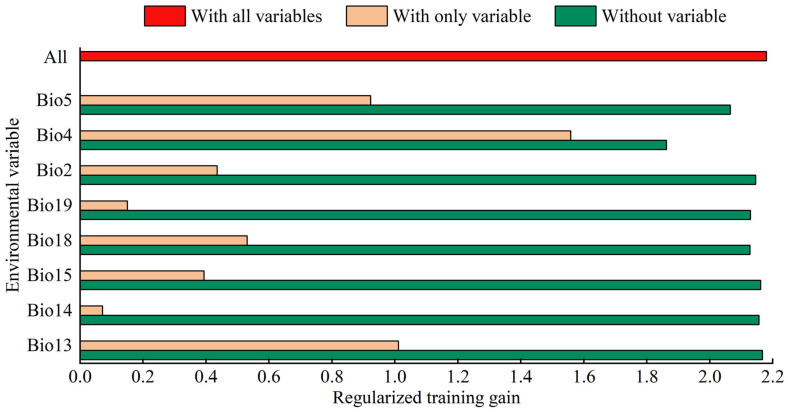
Jackknife test for bioclimatic variables of *Spodoptera exempta*. The red, yellowish, and green bars represent the total of all bioclimatic variables’ gain, gain for only one bioclimatic variable, and the total of all bioclimatic variables’ gain except this variable, respectively.

The top three bioclimatic variables that influenced potentially suitable areas for *S. exempta* were standard deviation temperature seasonality (Bio4: 62.5%), maximum temperature of the warmest month (Bio5: 14.2%), and precipitation of the coldest quarter (Bio19: 7.3%) (Figure S3). The jackknife analysis revealed that the standard deviation temperature seasonality (Bio4), precipitation of the wettest month (Bio13), and maximum temperature of the warmest month (Bio5) exhibited the three highest predictive factors (Figure 3). Overall, the key bioclimatic variables that influenced the potentially suitable areas for *S. exempta* included standard deviation temperature seasonality (Bio4), maximum temperature of the warmest month (Bio5), precipitation of the wettest month (Bio13), and precipitation of the coldest quarter (Bio19).

**Figure 4:**
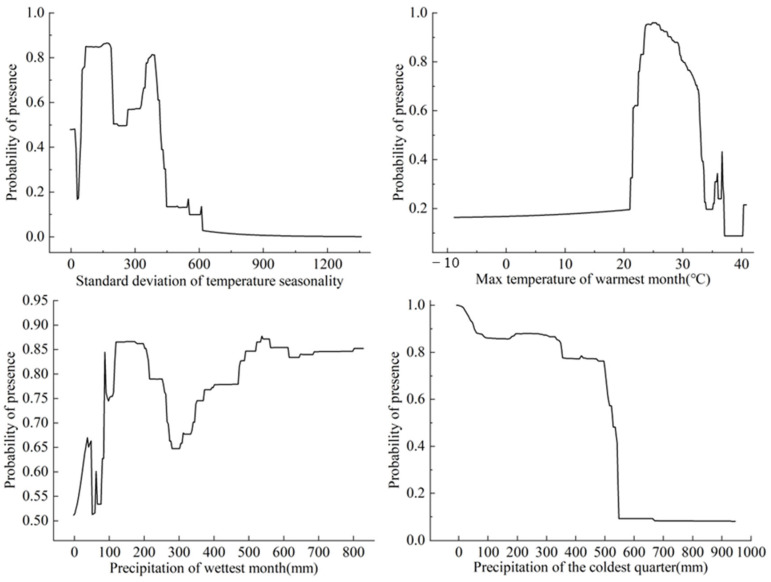
Response curves of the important bioclimatic variables of *Spodoptera exempta*: standard deviation temperature seasonality (Bio4); max temperature of warmest month (Bio5); precipitation of wettest month (Bio13); precipitation of coldest quarter (Bio19).

The individual response curves specific to each bioclimatic variable are shown in Figure 4. For *S. exempta*, the suitable ranges for Bio4, Bio5, Bio13, and Bio19 were 38.54–444.67, 14.79–36.94 °C, 0–866 mm, and 0–544.63 mm, respectively. When Bio4 exceeded 30.00, the probability of *S. exempta*’s presence sharply increased, peaking at 171.10, with the probability reaching as high as 0.86. Subsequently, the curve decreased sharply before surging to the second-highest probability (0.81). When Bio5 exceeded 21.07 °C, the survival probability of *S. exempta* increased sharply, reaching its peak at 24.78 °C with a survival probability of 0.96. The Bio13 curve remained relatively stable but rapidly increased between 301.37 and 558.67 mm. It peaked at 556.10 mm, reaching a probability of 0.87. In contrast, the Bio19 curves continuously declined after 496.48 mm, eventually stabilizing.

### 3.3. Potential Suitable Area of Spodoptera exempta under Current Climatic Conditions

**Figure 5:**
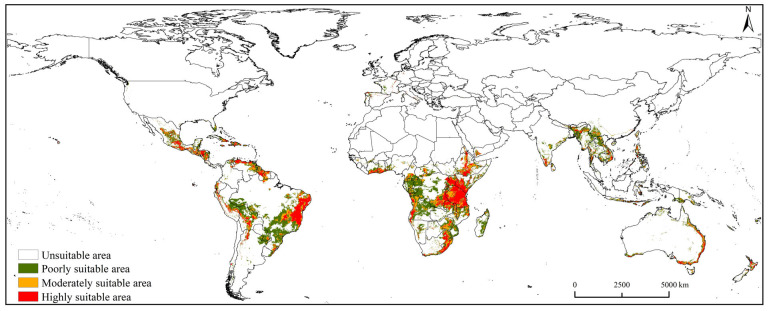
Global of potential suitable areas for *Spodoptera exempta* under current climatic conditions.

Figure 5 depicts the potential suitable areas for *S. exempta* under the current climatic conditions. The total suitable area was 1368.24 × 10^4^ km^2^, constituting approximately 10% of the global land mass, excluding Antarctica (Figure S4). *Spodoptera exempta* predominantly inhabited tropical and semiarid regions, including southern North America, central South America, southern Africa, southern Asia, and eastern Australia. The highly suitable area covered 325.37 × 10^4^ km^2^ (approximately 24% of the total suitable area), mainly distributed in eastern Brazil and eastern and southern Africa (Figure 5 and Figure S4). The moderately suitable area spanned 312.09 × 10^4^ km^2^ (approximately 23% of the total suitable area), mainly distributed in southern Africa, southeastern North America, central South America, and southern Asia. The poorly suitable area accounted for 730.78 × 10^4^ km^2^ (approximately 53% of the total suitable area), mainly distributed in central South America, southern Africa, and southern Asia.

### 3.4. Potential Suitable Area of Spodoptera exempta under Future Climatic Conditions

**Figure 6:**
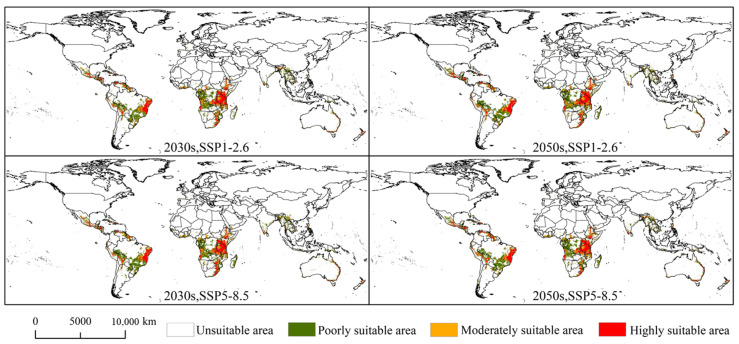
Global of potential suitable areas for *Spodoptera exempta* under future climatic conditions (SSP-2.6 and SSP5-8.5 in the 2030s and 2050s, respectively).

Figure 6 depicts the potential suitable areas for *S. exempta* under future climatic conditions. Overall, no significant difference emerged between the potential suitable areas for *S. exempta* under the current and future climatic conditions (Figure S4). Under future climate conditions, the largest highly suitable area is 305.65 × 10^4^ km^2^ at 2050s under SSP1-2.6, followed by 303.77 × 10^4^ km^2^ in the 2050s under SSP5-8.5, 301.69 × 10^4^ km^2^ in the 2030s under SSP5-8.5, and 288.33 × 10^4^ km^2^ in the 2030s under SSP1-2.6. The largest moderately suitable area is 298.22 × 10^4^ km^2^ in the 2030s under SSP5-8.5, followed by 296.21 × 10^4^ km^2^ in the 2050s under SSP1-2.6, 294.22 × 10^4^ km^2^ in the 2030s under SSP1-2.6, and 290 × 10^4^ km^2^ in the 2050s under SSP5-8.5. The largest lowly suitable area is 719.1 × 10^4^ km^2^ in the 2030s under SSP5-8.5, followed by 705.99 × 10^4^ km^2^ in the 2050s under SSP5-8.5, 684.43 × 10^4^ km^2^ in the 2050s under SSP1-2.6, and 678.58 × 10^4^ km^2^ in the 2030s under SSP1-2.6. The potential suitable areas for *S. exempta* under future climatic conditions exhibited some reduction compared to the current ones, indicating that global warming may limit the distribution of *S. exempta*.

### 3.5. Overlapping Areas of Spodoptera exempta and Global Wheat, Rice, and Maize Planting Areas

**Figure 7:**
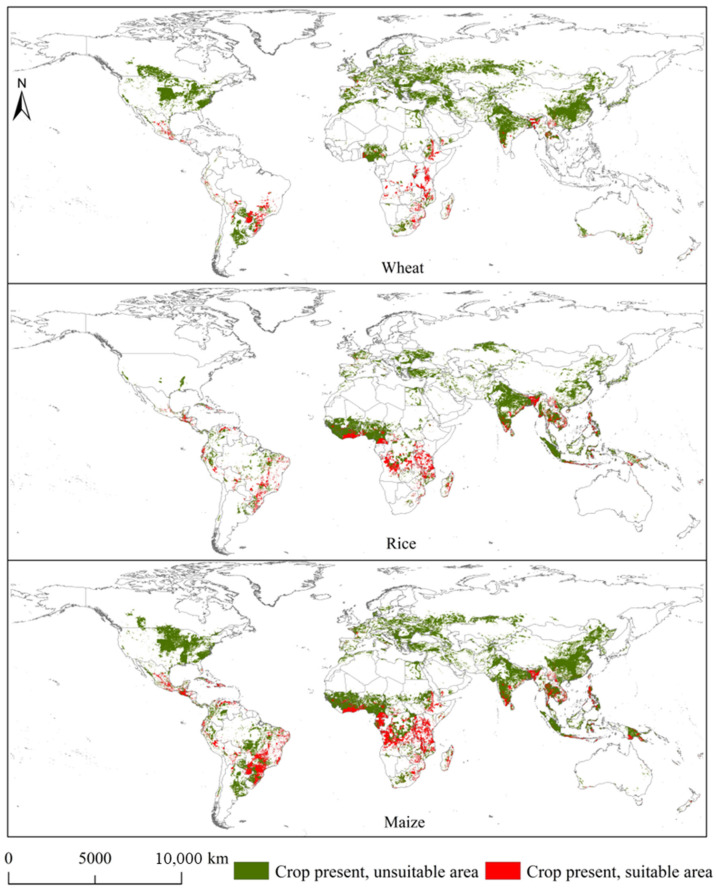
Overlapping areas of *Spodoptera exempta* and global wheat, rice, and maize planting areas under the current climatic conditions.

**Figure 8:**
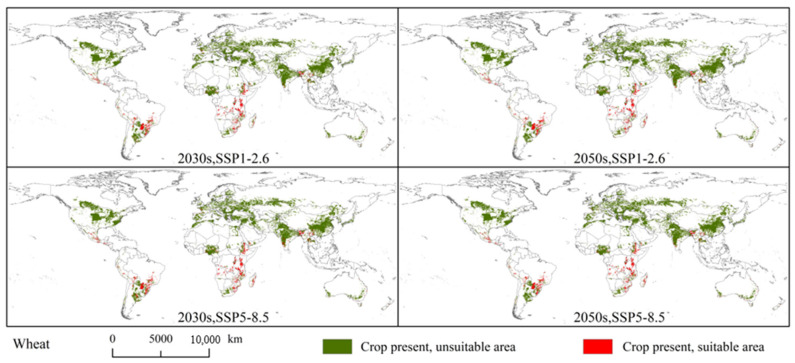
Overlapping areas of *Spodoptera exempta* and global wheat planting areas under the SSP1-2.6 and SSP5-8.5 in 2030s and 2050s.

**Figure 9:**
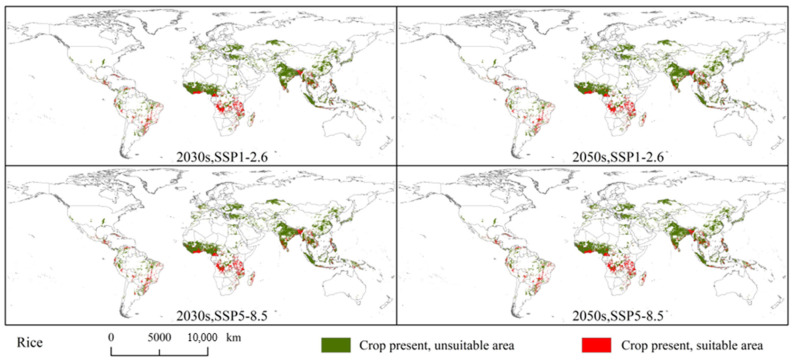
Overlapping areas of *Spodoptera exempta* and global rice planting areas under the SSP1-2.6 and SSP5-8.5 in 2030s and 2050s.

**Figure 10:**
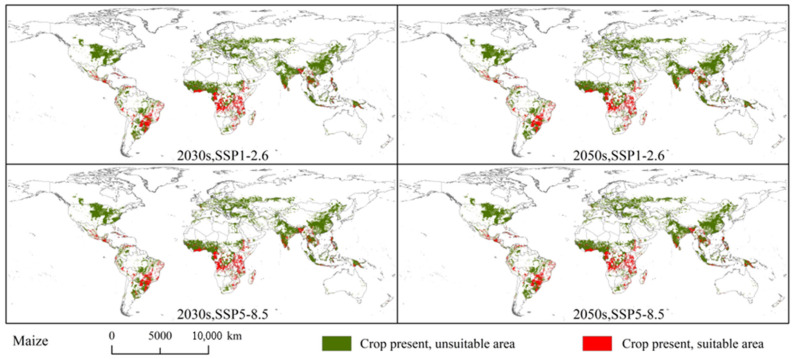
Overlapping areas of *Spodoptera exempta* and global maize planting areas under the SSP1-2.6 and SSP5-8.5 in the 2030s and 2050s.

Figure 7, Figure 8, Figure 9 and Figure 10 illustrates the overlapping areas between *S. exempta* and global wheat, rice, and maize planting areas under the current and future climatic conditions. Under the current climatic conditions, the overlap between the global wheat planting areas and potentially suitable areas for *S. exempta* covered 190.99 ×10^4^ km^2^ (approximately 10.0% of the global wheat planting area). There is no significant difference in the distribution of overlapping areas under the current and future climatic conditions. The overlapping areas are mainly located in central South America (southern Brazil, southern Paraguay, central Bolivia, and Peru), southern North America (central Mexico and Guatemala), central and southern Africa (Ethiopia, Kenya, Tanzania, Zimbabwe, South Africa, and Angola), southern Asia (southwestern China, Thailand, southern and northwestern India, Bangladesh), western Europe (Spain and France), and eastern Oceania (eastern Australia) (Figure 7 and Figure 8). The overlap area was largest under SSP5-8.5 in the 2030s (190.23 × 10^4^ km^2^), followed by SSP1-2.6 in the 2050s (181.44 × 10^4^ km^2^), SSP15-8.5 in the 2050s (180.19 × 10^4^ km^2^), and SSP1-2.6 in the 2030s (172.94 × 10^4^ km^2^) (Figure S5). Under future climate conditions, the overlap area will be somewhat reduced compared to that under the current climate conditions.

Under the current climatic conditions, the overlapping area between the global rice planting areas and potential suitable areas for *S. exempta* amounted to 312.3 × 10^4^ km^2^ (approximately 20.89% of the global rice planting area). There exists no significant difference in the distribution of overlapping areas under the current and future climatic conditions. The overlap areas are mainly located in southern North America (southern and central Mexico, Honduras, Guatemala, and Cuba), eastern South America (eastern Brazil, Bolivia, Peru, and southern Venezuela), central and southern Africa (Tanzania, Mozambique, Madagascar, Angola, Democratic Republic of the Congo, Cameroon, southern Ghana), and southern Asia (southern India, Burma, Vietnam, Thailand, southwest China, and Indonesia) (Figure 7 and Figure 9). The overlap area was largest under SSP5-8.5 in the 2050s (296.36 × 10^4^ km^2^), followed by SSP5-8.5 in the 2030s (295.83 × 10^4^ km^2^), SSP1-2.6 in the 2050s (288.03 × 10^4^ km^2^), and SSP1-2.6 in the 2030s (283.96 × 10^4^ km^2^) (Figure S5). Under future climatic conditions, the overlap area will be reduced to some extent.

Among the three crops, the global maize planting areas are most threatened by *S. exempta*. Under the current climatic conditions, the overlap between the global maize planting areas and potential suitable areas for *S. exempta* covered 510.78 × 10^4^ km^2^ (approximately 19.25% of the global maize planting area). There is no significant difference in the distribution of overlapping areas under current and future climatic conditions. The overlapping areas are mainly located in southern North America (central Mexico, Guatemala, Cuba), eastern South America (Central and southern Brazil, southern Paraguay, Bolivia, Peru, northern Venezuela), eastern and central Africa (Ethiopia, Kenya, Tanzania, Mozambique, Madagascar, South Africa, Zimbabwe, Angola, and Democratic Republic of the Congo), and southern Asia (southwestern China, southern India, Bangladesh, Vietnam, Myanmar, Philippines, and Indonesia) (Figure 7 and Figure 10). The overlapping area was largest under SSP5-8.5 in the 2030s (492.91 × 10^4^ km^2^), followed by SSP5-8.5 in the 2050s (490.27 × 10^4^ km^2^), SSP1-2.6 in the 2050s (479.83 × 10^4^ km^2^), and SSP1-2.6 in the 2030s (479.29 × 10^4^ km^2^) (Figure S5). Under future climatic conditions, the overlap area will also be reduced to some extent.

## 4. Discussion

*Spodoptera exempta* is a prevalent agricultural pest that presents a serious threat to major crops worldwide, such as maize, wheat, and rice [5]. However, the worldwide potential geographic distribution of *S. exempta* and its damage to maize, wheat, and rice are currently unknown. Here, we studied the potential geographic distribution of *S. exempta* and its overlap with the three crops’ planting areas under the current and future climatic conditions using the optimized MaxEnt model. Our research provides theoretical guidance for *S. exempta* control and for the conservation of major crops worldwide.

### 4.1. Model Accuracy Assessment

Ecological niche models have been widely used to predict the geographic distribution of species, but ensuring the accuracy of predictions requires consideration of multiple factors during the modeling process. Due to the absence of biological parameters of the species, to ensure the accuracy of the prediction results, we chose the correlation model (MaxEnt). Species distribution data and environmental variables determine the accuracy of the model, so wherever possible, high-quality distribution data and environmental variables closely associated with species distribution were collected [30]. Meanwhile, in order to avoid overfitting the model, we cleaned the distribution data and filtered the environmental variables, respectively [28,31]. The FC and RM of the model were also optimized using the ENMeval package, and the results also showed better prediction than the default parameter model [27]. The value of the mean AUC was 0.964, while the prediction may also overlap with the actual distribution of *S. exempta*. Therefore, we believe that the predictions of the model are reliable.

### 4.2. Important Bioclimatic Variables Affecting the Geographic Distribution Patterns of Spodoptera exempta

Climate change significantly influences the potential distribution of agricultural invasive pests [32]. Gómez-Undiano et al. predicted the global potential suitable areas for *S. exempta* using the Biomod2 platform and found that annual precipitation, annual mean temperature, and isothermality were the major predictors for determining the potential suitable areas for *S. exempta* [7]. Our results showed that the potential suitable areas for *S. exempta* are associated with temperature and precipitation, especially standard deviation temperature seasonality, max temperature of the warmest month, precipitation of the wettest month, and precipitation of the coldest quarter, which would play critical roles in the potential distribution. Moreover, in varying environmental conditions, solitary larvae exhibit higher survival rates in high temperatures, while gregarious larvae tend to thrive better in lower temperature environments. During the first two months after the beginning of the short rainy season, the number of solitary larvae of *S. exempta* decrease with the decrease in temperature; however, for gregarious larvae, a decrease in temperature leads to an increase in population size [33]. Furthermore, the rainy season is very important for the movement of *S. exempta* adults because wind, which mainly occurs during the rainy season, is key to adult dispersal. Adults migrate along the wind direction to grassland or crop areas where they feed, leading to subsequent infestation with larvae in nearby areas [34,35,36]. Overall, previous studies verified the results of our study.

### 4.3. The Potential Suitable Areas of Spodoptera exempta in the World under Current and Future Climatic Conditions

Our results indicate that under the current climatic conditions the potential suitable areas for *S. exempta* are mainly located in tropical and semiarid areas, including southern North America, central South America, central Africa, southern Asia, and eastern Australia. Furthermore, the overlap between these suitable areas for *S. exempta* and global crop production areas (wheat, rice, and maize) exposes different countries to varying levels of risk. Specifically, concerning wheat planting areas, southern Brazil, southern Paraguay, central Bolivia, Peru, central Mexico, Guatemala, Ethiopia, Kenya, Tanzania, Zimbabwe, South Africa, Angola, southwestern China, Thailand, southern, northwestern India, Bangladesh, Spain, France, and eastern Australia faced higher susceptibility to invasive threats posed by *S. exempta*. Considering overlap with rice planting areas, southern and central Mexico, Honduras, Guatemala, Cuba, eastern Brazil, Bolivia, Peru, southern Venezuela, Tanzania, Mozambique, Madagascar, Angola, Democratic Republic of the Congo, Cameroon, southern Ghana, southern India, Burma, Vietnam, Thailand, southwest China, and Indonesia are at higher risk. Considering overlap with maize planting areas, the areas at higher risk are nearly consistent with the potential suitable areas for *S. exempta*, that is, southern countries of North America, central and eastern countries of South America, central and southern countries of Africa, and southern countries of Asia. In particular, the United States is the center of diversity and a native region of maize, and even though *S. exempta* does not have a potential distribution area here, there is one nearby in Mexico, so we believe that the species poses a similarly high risk of harm to maize planting areas in the United States. Of the three major crops, wheat, rice, and maize, the potential threat to maize planting areas worldwide is potentially the greatest. Previous studies have also indicated that *S. exempta* is a major pest to maize crops in eastern and southern Africa [37]. Meanwhile, in the laboratory, *S. exempta* can be reared very successfully on a diet containing dried maize leaf meal [38]. These findings align with the conclusions drawn in this study. Moreover, the high potential threat from *S. exempta* extends to all three major crops in several countries, including Mexico, Brazil, Bolivia, Paraguay, Uruguay, Ethiopia, South Africa, Zimbabwe, Myanmar, Australia, India, and China. In conclusion, our study showed a slight reduction in the area of potential suitable areas for *S. exempta* under future climate change conditions, but the three main planting areas of wheat, rice, and maize, which still significantly overlap with the potential suitable areas for *S. exempta*, should all be of sufficient concern, especially in maize planting areas.

### 4.4. Prevention and Control Measures of Spodoptera exempta

Early warning is more important than control for invasive alien species [39]. Our results showed that, under the current climatic conditions, *S. exempta* has a wide distribution of potential suitable areas and overlapping crop planting areas in South and North America, despite there being no occurrence records of the species available for these areas. However, these areas are similar to the actual distribution areas of the species in climatic conditions, and there is significant trade between the regions, and if *S. exempta* was introduced into these areas, it would spread rapidly and pose a great risk to food security [7]. Therefore, these areas should strengthen their quarantine efforts to prevent the invasion of *S. exempta*. At the same time, African countries should strengthen the control and management of *S. exempta*, which we consider to be native based on the time the species was studied and its presence in Africa, although there are no clear studies indicating the native range of the species. The mode of spread and outbreak of *S. exempta* is mainly migration flying [40]. In particular, prevention and control should be intensified during the annual rainy season because studies have proven that the winds generated during the rainy season are key to the spread of *S. exempta* [7]. During severe outbreaks, *S. exempta* can migrate windward for up to 100 km or more; therefore, widespread monitoring and prevention is needed [41]. Meanwhile, when *S. exempta* individuals are encountered, reasonable measures should be taken to eradicate them on time, including cultural control, biological control and chemical control measures. Cultural control focuses on removing weeds near crops because crops over 50 cm are less likely to be damaged by the larvae of *S. exempta*, which can subsequently damage crops if they develop weeds [40]. Biological control methods have gained attention, with the nuclear polyhedrosis virus (SpexNPV) being utilized to manage outbreaks of *S. exempta* [37]. Chemical control involves the use of insecticides to eliminate the larvae of *S. exempta*, because the larvae are sensitive to multiple insecticides and no resistance has been recorded. These insecticides, classified as organophosphorus compounds, carbamates, and synthetic pyrethroids, exhibit greater effectiveness, particularly when applied after heavy rain [42]. In summary, countries should build up a complete system for early warning, quarantine, and prevention to effectively control *S. exempta* to protect global food security.

Under future climatic conditions, we used data from current host crop planting areas overlayed with future suitable areas for *S. exempta* to show the areas where the species is harming crops. This is due to the lack of future data, and subsequent studies will need to focus on crop planting area data for future periods.

## 5. Conclusions

We predicted the global potential suitable area of *S. exempta* using an optimized MaxEnt model, and the results were overlapped with global wheat, rice, and maize planting areas. The optimized model for predicted *S. exempta* has high reliability in the global potential suitable areas. Temperature has a stronger effect on the global distribution of this species than precipitation. The standard deviation temperature seasonality (Bio4) and max temperature of the warmest month (Bio5) are the most two important bioclimatic variables influencing on the global distribution of *S. exempta*. Under the current climatic conditions, Latin America, Central Africa, and Southeast Asia, which are the areas of concentration for developing countries, were mainly areas of high-risk distribution of *S. exempta*, while global warming would have some limitations on the global geographic distribution. The global potential suitable areas for the species would not expand further in the future. Overall, *S. exempta* had the greatest impact on global planting areas of maize, mainly in southern North America, eastern South America, central and eastern Africa, and southern Asia; the second greatest impact was on the global planting areas of rice, mainly in the southern North America, eastern South America, central and southern Africa, and southern Asia; and the smallest impact was on the global planting areas of wheat, mainly in central South America, southern North America, central and southern Africa, and southern Asia. Global warming will limit the impact of *S. exempta* on the three major crops. Under future climate scenarios, damage caused by *S. exempta* to the three crops’ planting areas will be somewhat reduced compared to that in the current period. Our study provides a theoretical reference for the global quarantine sector, especially for developing countries, to prevent and control *S. exempta*, thus protecting global food security.

## Data Availability

Raw data can be obtained from the corresponding author.

## Supplementary Materials

The following supporting information can be downloaded at: https://www.mdpi.com/article/10.3390/insects15050348/s1, Table S1: Meanings of 19 bioclimatic variables in worldclim; Table S2: Meanings of the selected two emission scenarios; Figure S1: Eight bioclimatic variables used to construct the MaxEnt model: absolute correlations were less than 0.8 (|r| < 0.8); Figure S2: ROC curve and the value of Mean (AUC) for the MaxEnt model after optimal parameter combination; Figure S3: Contribution of each bioclimatic variables related to the distribution of *Spodoptera exempta*; Figure S4: The change of the potential suitable area of *Spodoptera exempta* under current and future climatic conditions; Figure S5: The change of the overlapping areas of *Spodoptera exempta* intersected with global wheat rice and maize acreage under current and future climatic conditions.
