# Supplementary material for: Risk Assessment of Spodoptera exempta against Food Security: Estimating the Potential Global Overlapping Areas of Wheat, Maize, and Rice under Climate Change
Source: Insects. 2024 May 13;15(5):348. doi: 10.3390/insects15050348 (PMC11121843; doi:10.3390/insects15050348)
Supplement: Supplementary file 1 [file insects-15-00348-s001.zip › insects-2964751-supplementary.pdf]

Supplementary material

Table S1. Meanings of 19 bioclimatic variables in worldclim

Variable	Description	Unit
Bio1	Annual mean temperature	℃
Bio2	Mean diurnal air temperature range	℃
Bio3	Isothermality (bio2/bio7) (*100)	-
Bio4	Standard deviation temperature seasonality	-
Bio5	Max temperature of warmest month	℃
Bio6	Min temperature of coldest month	℃
Bio7	Temperature annual area (bio5-bio6)	℃
Bio8	Mean temperature of wettest quarter	℃
Bio9	Mean temperature of driest quarter	℃
Bio10	Mean temperature of warmest quarter	℃
Bio11	Mean temperature of coldest quarter	℃
Bio12	Annual precipitation	mm
Bio13	Precipitation of wettest month	mm
Bio14	Precipitation of driest month	mm
Bio15	Precipitation seasonality (coefficient of variation)	-
Bio16	Precipitation of wettest quarter	mm
Bio17	Precipitation of driest quarter	mm
Bio18	Precipitation of warmest quarter	mm
Bio19	Precipitation of coldest quarter	mm

Table S2. Meanings of the selected two emission scenarios.

Emission	Description
SSP1-2.6	SSP1 (Low forcing scenario) Upgrade to RCP2.6 scenario based on (Radiative forcing reaches 2.6 W/m^2 in 2100)
SSP5-8.5	SSP5 (High Forcing Scenario) Upgrade to RCP8.5 scenario based on (SSP5 is the only SSP scenario that can achieve radiative forcing to 8.5 W/m^2 in 2100)

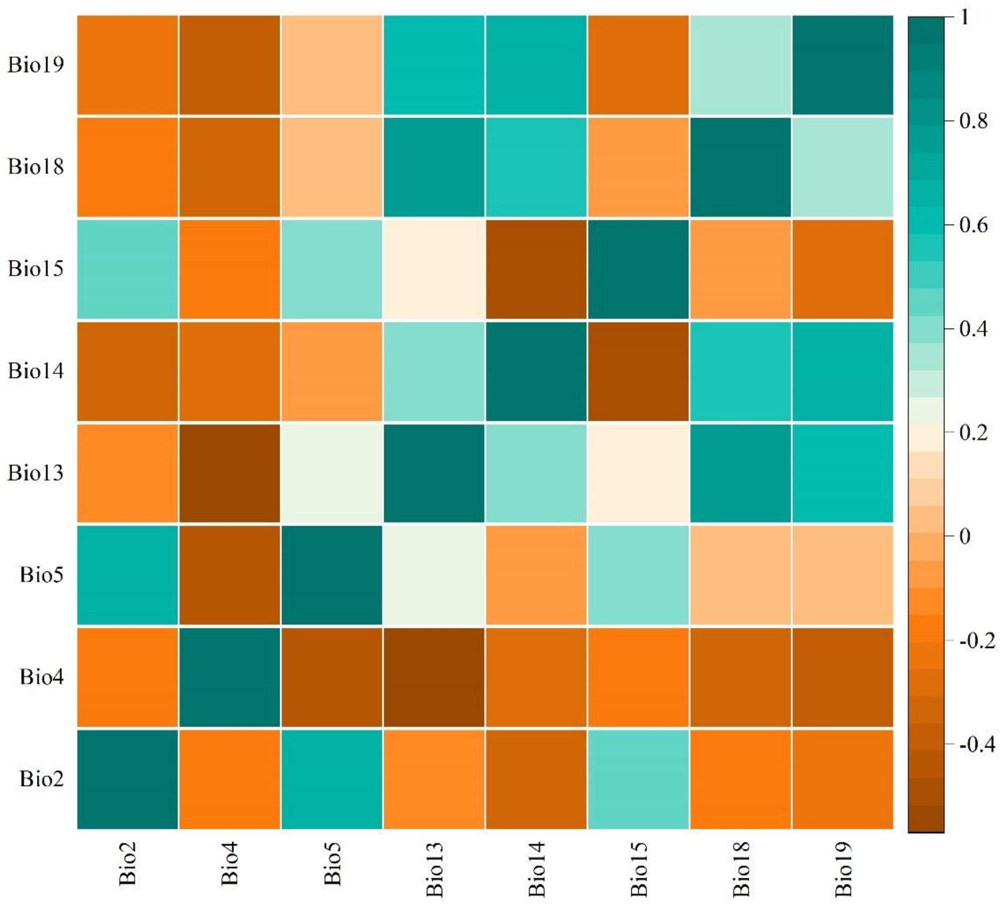

Figure S1. Eight bioclimatic variables used to construct the MaxEnt model: absolute correlations were less than 0.8 (|r|<0.8).

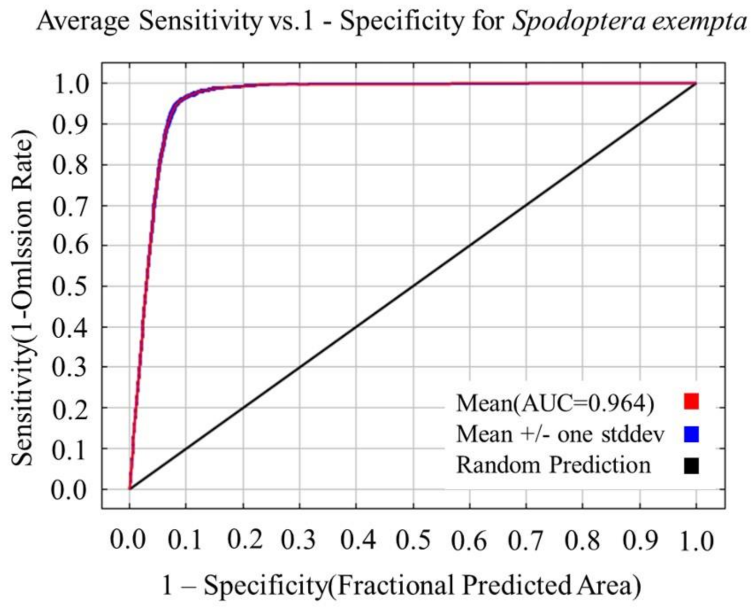

Figure S2. ROC curve and the value of Mean (AUC) for the MaxEnt model after optimal parameter combinations.

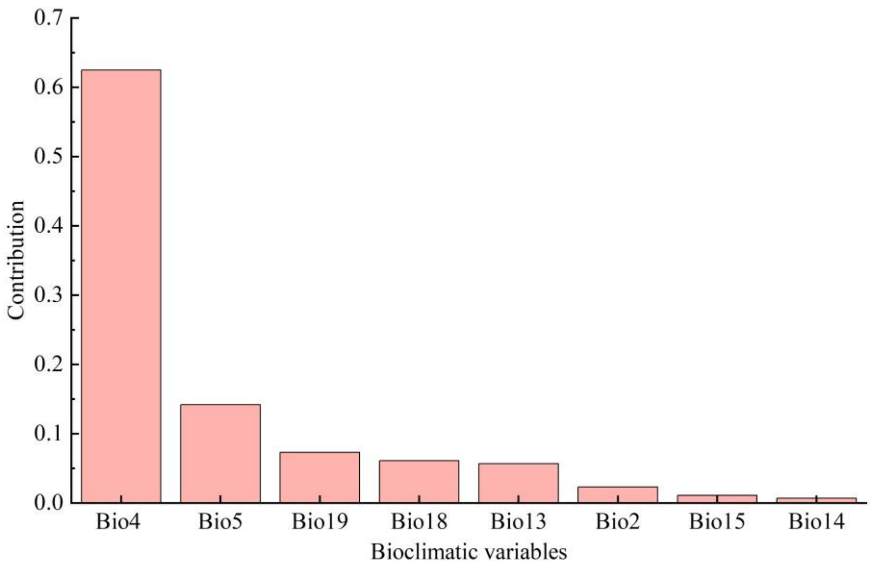

Figure S3. Contribution of each bioclimatic variables related to the distribution of *Spodoptera exempta*.

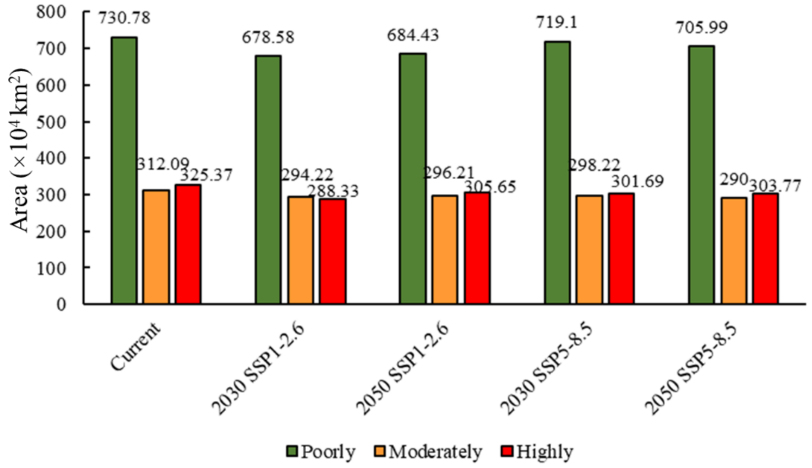

Figure S4. The change of the potential suitable area of *Spodoptera exempta* under current and future climatic conditions

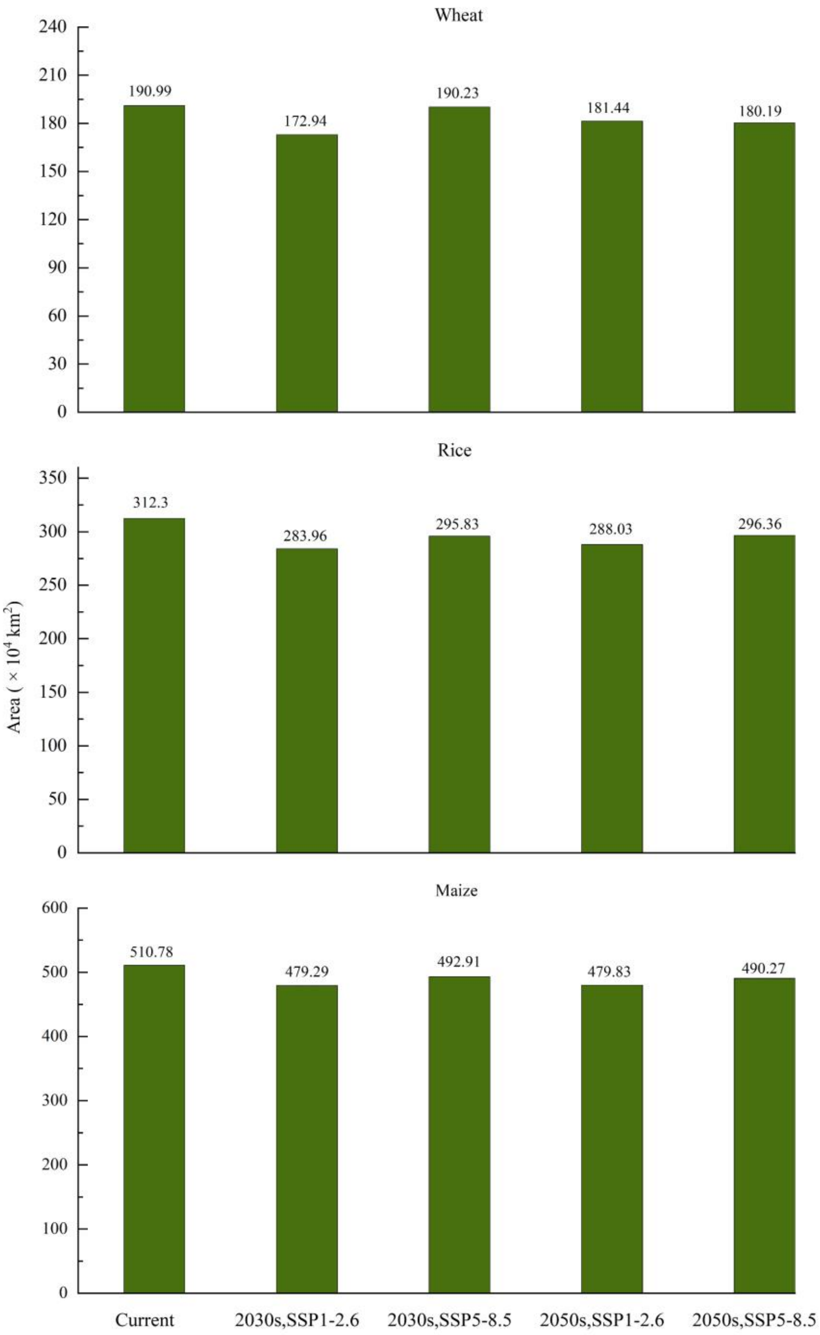

Figure S5. The change of the overlapping areas of *Spodoptera exempta* intersected with global wheat, rice and maize acreage under current and future climatic conditions.
